# Supplementary material for: Transglutaminase 2 function in glioblastoma tumor efferocytosis
Source: Cell Death Dis. 2025 Jul 3;16(1):487. doi: 10.1038/s41419-025-07819-2 (PMC12229499; doi:10.1038/s41419-025-07819-2)
Supplement: Supplementary file 2 — Supplementary Table 1 [file 41419_2025_7819_MOESM2_ESM.pdf]

Supplementary Table 1

TMAs with duplicate IDH wild type glioblastoma patient samples were stained for TGM2 expression. Individual cores were scored for: no positive staining (“all negative”); cancer cells positive (“uniform positive cancer cell”); endothelial cells present and positive (”endothelial”); occasional individual positive cells, likely macrophages (sporadic positive cells); cluster of positive cells (“focal positive”); whether necrosis was observed (“necrosis present”). Samples were across four TMA slides (TMAs 8, 9, 10 and 11). Cases for which only one sample was present are highlighted in grey. 5 % of cores (7/152) showed no staining; 3 % of cores (5/152) showed uniform positive cancer cells staining; 73 % of cores (111/152) showed positive endothelial cell staining; 84 % of cores (127/152) showed sporadic positive cells; 17 % of cores (26/152) showed focal positive staining; 3 % of cores (5/152) showed necrosis (four of these were also focal positive for TGM2).

	TMA #8 SAMPLE ID	all negative	uniform positive cancer cell	endothelial	sporadic positive cells	focal positive	necrosis present
1	S18-11039 B		n	y	y	n	n
2	S18-11039 B		n	n	y	n	?
3	S18-11075 2A		n	y	y	n	n
4	S18-11075 2A		n	n	y	n	n
5	S18-11377 8B		n	y	n	n	n
6	S18-11377 8B		n	y	n	n	n
7	S18-12174 A		n	y	y	y	y
8	S18-12174 A		n	y	y	n	n
9	S18-13439 2C		n	y	y	n	n
10	S18-13439 2C		n	y	y	n	n
11	S18-16928 2A		n	y	y	y	n
12	S18-1821 2A		n	y	y	n	n
13	S18-1821 2A		n	y	y	y	n
14	S18-19332 2A		n	y	y	n	n
15	S18-19332 2A		n	y	n	n	n
16	S18-2862 D		n	y	y	y	y
17	S18-2862 D		n	y	y	n	n
18	S18-3314 2A		n	y	y	n	n
19	S18-3314 2A		n	y	y	y	n
20	S18-5272 A		n	y	y	n	n
21	S18-5272 A		n	y	y	y	?
22	S19-10153 A	y					
23	S19-10153 A	y					
24	S19-3207 C		n	y	y	n	n
25	S19-3207 C		n	y	y	n	n
26	S19-4196 2A		n	y	y	n	n
27	S19-4196 2A		n	y	n	n	n
28	S19-6017 C		n	y	n	n	n
29	S19-6017 C		n	y	y	n	n
30	S19-6232 1B		n	y	n	n	n
31	S19-6232 1B		n	y	n	n	n
32	S19-688 2B		y?	y	y	n	n
33	S19-688 2B		y?		y	y	n
34	S19-7928 3C		n	y	y	n	n
35	S19-7928 3C		n	y	y	n	n
36	S19-901 B		y	y	y	n	n
37	S19-901 B	y					
38	S19-9289 2A		n	y	y	n	n
39	S19-9289 2A		n	y	y	n	n
40	S19-9405 4A		n	y	y	n	n
41	S19-9405 4A	y					

	TMA #9 SAMPLE ID	all negative	uniform positive cancer cell	endothelial	sporadic positive cells	focal positive	necrosis present
1	S18-1004 2A		n	y	y	y	n
2	S18-10905 A		n	y	y	n	n
3	S18-10905 A		n	y	y	n	n
4	S18-12115 A		n	n	y	n	n
5	S18-12339 2A		n	y	y	n	n
6	S18-12339 2A		n	n	y	n	n
7	S18-12553 2A		n	y	y	n	n
8	S18-12553 2A		n	n	y	y	?
9	S18-13375 2B		n	y	y	n	n
10	S18-13375 2B		n	y	y	n	n
11	S18-13681 2A		n	y	y	n	n
12	S18-13681 2A		n	y	y	n	n
13	S18-13710 2B		n	n	y	n	n
14	S18-13710 2B		n	n	y	n	n
15	S18-14101 A		n	n	y	n	n
16	S18-17530 2A		y?	n	y	y	n
17	S18-17530 2A		n	n	y	y	n
18	S18-1910 2A		n	y	y	n	n
19	S18-1910 2A		n	y	y	n	n
20	S18-19882 A		n	y	y	n	n
21	S18-19882 A		n	y	y	n	n
22	S18-20382 2B		n	y	y	n	n
23	S18-20382 2B		n	n	y	n	n
24	S18-21383 2A		n	y	y	n	n
25	S18-21731 5A		n	y	y	y	n
26	S18-21731 5A		n	n	y	n	n
27	S18-22238 2B		n	y	y	n	n
28	S18-22238 2B		n	y	y	n	n
29	S18-22460 2A		n	n	y	n	n
30	S18-22460 2A		n	y	y	n	n
31	S18-22710 4B		n	y	y	n	n
32	S18-22710 4B		n	y	y	n	n
33	S18-7198 3A	y					
34	S18-7198 3A	y					
35	S18-8889 6A		n	y	y	n	n
36	S18-8889 6A		n	y	y	n	n
37	S18-9448 A		n	n	y	y	n

	TMA #10 SAMPLE ID	all negative	uniform positive cancer cell	endothelial	sporadic positive cells	focal positive	necrosis present
1	S17-12038 2B		n	y	y	n	n
2	S17-12038 2B		n	y	y	n	n
3	S17-1371 13B		n	y	y	n	n
4	S17-1371 13B		n	y	y	n	n
5	S17-13731 B		n	y	y	y	y
6	S17-13731 B		n	y	y	y	y
7	S17-14933 1A		n	y	y	n	n
8	S17-14933 1A		n	y	y	n	n
9	S17-2343 2A		n	y	y	n	n
10	S17-2343 2A		n	n	y	n	n
11	S17-3905 2A		n	n	y	y	n
12	S17-4294 B		n	y	y	y	n
13	S17-4294 B		n	y	y	n	n
14	S17-4439 A		n	y	n	n	n
15	S17-5356 2A	y					
16	S17-5356 2A		n	n	y	n	n
17	S17-7262 3C		n	y	y	y	n
18	S17-7262 3C		n	y	y	n	n
19	S17-7311 A		n	n	y	y	n
20	S17-8524 B		n	n	n	n	n
21	S17-94 2B		y?	y	y	n	n
22	S17-94 2B		n	n	y	n	n
23	S18-12176 B		n	y	y	n	n
24	S18-12176 B		n	y	y	n	n
25	S18-12610 2A		n	y	n	n	n
26	S18-12610 2A		n	y	n	n	n
27	S18-15965 2A		n	n	y	n	y?
28	S18-15965 2A		n	n	y	n	n
29	S18-17755 2A		n	y	n	n	n

	TMA #11 SAMPLE ID	all negative	uniform positive cancer cell	endothelial	sporadic positive cells	focal positive	necrosis present
1	S17-10103 C		n	y	y	n	n
2	S17-10103 C		n	y	y	y	n
3	S17-10305 2B		n	y	y	n	n
4	S17-10305 2B		n	y	y	n	n
5	S17-10838 2B		n	y	y	n	n
6	S17-10838 2B		n	y	y	n	n
7	S17-11005 A		n	y	y	n	n
8	S17-11005 A		n	y	n	n	n
9	S17-11009 B		n	n	y	y	n
10	S17-11009 B		n	y	y	n	n
11	S17-11016 C		n	y	y	y	n
12	S17-11023 2A		n	n	y	n	n
13	S17-11235 2A		n	n	y	n	n
14	S17-11235 2A		n	y	y	n	n
15	S17-12255 9A		n	y	y	n	n
16	S17-12255 9A		n	y	y	n	n
17	S17-12835 2B		n	n	y	y	n
18	S17-12835 2B		n	y	n	n	n
19	S17-14567 3A		n	y	y	n	n
20	S17-14567 3A		n	y	y	n	n
21	S17-17332 2C		n	y	y	n	n
22	S17-17332 2C		n	y	y	n	n
23	S17-17437 13B		n	n	y	y	n
24	S17-17437 13B		n	n	y	y	n
25	S17-17457 19A		n	n	y	y	n
26	S17-17837 2B		n	y	y	n	n
27	S17-17837 2B		n	y	y	n	n
28	S17-18245 B		n	y	y	n	n
29	S17-18245 B		n	y	y	n	n
30	S17-18330 B		n	y	y	y	n
31	S17-18756 3A		n	n	y	n	n
32	S17-18756 3A		n	n	y	n	n
33	S17-19039 A		n	n	y	n	n
34	S17-19056 2A		n	y	y	n	n
35	S17-19056 2A		n	y	n	n	n
36	S17-20127 A		n	y	y	n	n
37	S17-20127 A		n	y	y	n	n
38	S17-20984 2C		n	y	n	n	n
39	S17-21863 2A		n	y	n	n	n
40	S17-21863 2A		n	y	y	n	n
41	S17-22098 A		n	y	y	n	n
42	S17-22098 A		n	y	y	n	n
43	S17-22218 1B		n	y	n	n	n
44	S17-9675 2A		n	n	y	n	n

45	S17-9675 2A	n	y	y	n	n
