# Supplementary figures and images for: Transglutaminase 2 function in glioblastoma tumor efferocytosis
Source: Cell Death Dis. 2025 Jul 3;16(1):487. doi: 10.1038/s41419-025-07819-2 (PMC12229499; doi:10.1038/s41419-025-07819-2)

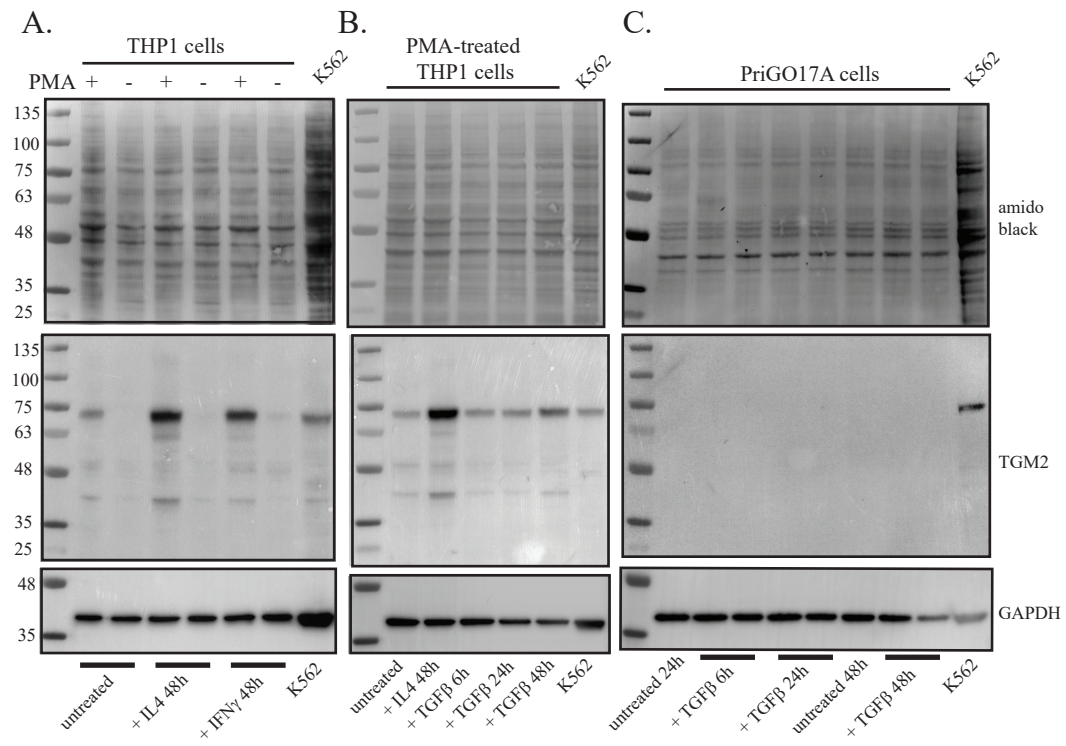

Supplement: Supplementary file 4 — Original data (same as Figure S2) [file 41419_2025_7819_MOESM4_ESM.pdf]
